# Phonological Working Memory Representations in the Left Inferior Parietal Lobe in the Face of Distraction and Neural Stimulation

**DOI:** 10.3389/fnhum.2022.890483

**Published:** 2022-06-23

**Authors:** Qiuhai Yue, Randi C. Martin

**Affiliations:** ^1^Department of Psychological Sciences, Rice University, Houston, TX, United States; ^2^Department of Psychology, Vanderbilt University, Nashville, TN, United States

**Keywords:** phonological working memory, supramarginal gyrus, buffer, functional magnetic resonance imaging, representational similarity analysis, distractor, transcranial magnetic stimulation

## Abstract

The neural basis of phonological working memory (WM) was investigated through an examination of the effects of irrelevant speech distractors and disruptive neural stimulation from transcranial magnetic stimulation (TMS). Embedded processes models argue that the same regions involved in speech perception are used to support phonological WM whereas buffer models assume that a region separate from speech perception regions is used to support WM. Thus, according to the embedded processes approach but not the buffer approach, irrelevant speech and TMS to the speech perception region should disrupt the decoding of phonological WM representations. According to the buffer account, decoding of WM items should be possible in the buffer region despite distraction and should be disrupted with TMS to this region. Experiment 1 used fMRI and representational similarity analyses (RSA) with a delayed recognition memory paradigm using nonword stimuli. Results showed that decoding of memory items in the speech perception regions (superior temporal gyrus, STG) was possible in the absence of distractors. However, the decoding evidence in the left STG was susceptible to interference from distractors presented during the delay period whereas decoding in the proposed buffer region (supramarginal gyrus, SMG) persisted. Experiment 2 examined the causal roles of the speech processing region and the buffer region in phonological WM performance using TMS. TMS to the SMG during the early delay period caused a disruption in recognition performance for the memory nonwords, whereas stimulations at the STG and an occipital control region did not affect WM performance. Taken together, results from the two experiments are consistent with predictions of a buffer model of phonological WM, pointing to a critical role of the left SMG in maintaining phonological representations.

## Introduction

Verbal working memory (WM) storage (also known as short-term memory, STM) refers to the capacity of maintaining verbal information in an accessible format to support cognitive operations and the planning of behavioral responses. A failure of maintaining verbal WM representations (e.g., due to damaged neural substrates of verbal WM) would impair subsequent behavioral performance (e.g., reducing recall of memory items). Thus, an important property of the verbal WM store is to prevent memory representations from being degraded by task-irrelevant interference coming from internal or external sources. To date, the theoretical basis and neural loci of verbal WM storage are still under debate.

At a theoretical level, embedded processes models claim that WM consists of the activated portion of long-term memory (LTM; Oberauer and Lange, 2009; Cowan et al., 2021); thus, WM is assumed to recruit the same brain regions which are involved in processing a specific type of information (Jonides et al., 2005; Postle, 2006). The lateral superior temporal gyrus (STG; particularly on the left) is involved in the processing of speech through which low-level acoustic representations are mapped onto long-term memory representations for phonological features (Turkeltaub and Coslett, 2010; Price, 2012; Yi et al., 2019). According to the embedded processes models, such temporarily activated phonological representations in the left STG constitute verbal WM. If the left STG serves as the sole neural substrate of short-term maintenance of phonological information, the disruption of memory representations maintained in this region (e.g., by either task-irrelevant verbal distractors or external neural stimulation) would cause a reduction in verbal WM performance. If successful WM performance is achieved despite this interference, such would suggest a separate module other than the processing system that is capable of temporarily housing WM representations while the speech processing system continues to process up-coming stimuli that are irrelevant to WM performance. Such a module has been conceptualized in multi-component buffer models of WM. These models propose dedicated temporary stores (i.e., buffers) for different types of information (e.g., visual-spatial vs. phonological), and these stores are different from long-term memory (LTM) or processing systems (e.g., visual and speech perception systems) in that domain (Baddeley et al., 2021; Martin et al., 2021a; Purcell et al., 2021). According to buffer accounts, once representations have been transferred into the buffer, the processing of distractors in perceptual regions would not disrupt WM performance (Xu, 2017, 2018), and interference would occur only if the representations held in a buffer were disturbed (e.g., by external neural stimulation). A way of addressing these claims is to test the causal role of the speech processing region and the buffer region in verbal WM. Neuropsychological studies with brain-damaged patients have provided evidence bearing on this issue, showing that an impairment of the left inferior parietal lobe (particularly the ventral part of the left supramarginal gyrus, SMG) was associated with deficits in verbal WM (Baldo and Dronkers, 2006; Paulesu et al., 2017; Martin et al., 2021b; Purcell et al., 2021). This inferior parietal lobe region is different from the speech perception region in the STG^1^ and has been proposed as the neural substrate of a phonological buffer (Martin, 2005). However, some studies using lesion-symptom mapping approaches have reported an association between the degree of damage in the left STG and phonological WM performance, suggesting that the left STG serves as the neural substrate of phonological WM (e.g., Leff et al., 2009; Baldo et al., 2012), but these studies have limitations. For example, in Leff et al. (2009) study, performance on a nonword repetition task was partialled out in the lesion-behavior correlational analyses. Nonword repetition has been argued to reflect an important component of phonological WM (Gupta, 2003; Majerus, 2013). In Baldo et al. (2012) study, speech perception abilities were not controlled for. In a recent study in our lab, when these issues were addressed, the lesions associated with impaired phonological WM capacity were primarily localized in the left SMG (Martin et al., 2021b). Neuroimaging work with healthy subjects also found mixed evidence regarding the neural substrate of phonological WM. Some studies found that the speech processing regions showed neural evidence for phonological WM (e.g., Ravizza et al., 2011; also see Buchsbaum and D’Esposito, 2008), though in those studies the speech processing region in the left STG was not well defined. In a recent study, using an independent localizer task involving syllable discrimination, we defined the phonological processing region in the left STG but did not find significant neural evidence for phonological WM in this region. Instead, consistent evidence for a phonological WM buffer in the left SMG was found (Yue et al., 2019). One means of providing converging evidence with healthy subjects is to apply neural stimulation (e.g., transcranial magnetic stimulation, TMS) to the SMG to temporarily disturb neural activity at this putative buffer region (Cohen et al., 1997; Pascual-Leone et al., 1999) and determine how behavioral performance is affected. TMS is a noninvasive technique of brain stimulation which uses an electromagnetic coil that is placed on the scalp to induce electric current applied to a specific brain region *via* electromagnetic induction. The induced current has been assumed to produce either excitatory or inhibitory effects on the neuronal activity of the stimulated area, depending on its intensity and frequency (Hallett, 2007; Valero-Cabré et al., 2017; Pitcher et al., 2021). Previous studies using either single-pulse, triple-pulse, or repetitive TMS procedures have been shown to disrupt WM functions (e.g., Oliveri et al., 2001; Desmond et al., 2005; also see a detailed discussion in the introduction to the TMS experiment). Thus, testing distractor and neural stimulation interference effects and examining their neural loci provides a means of evaluating the theoretical debate of embedded processes vs. buffer accounts of verbal WM by determining whether it is the processing region or the buffer region that plays an essential role in WM storage.

In the current study, we carried out two experiments with neuroimaging and brain stimulation approaches to examine the neural locus for phonological WM storage and test its resistance to inference. The first was an fMRI experiment in which we used a representational similarity analysis (RSA) approach which explicitly modeled phonological WM representations for individual items (Yue and Martin, 2021) during the delay period of a recognition memory task. The task included a distractor manipulation with distractors presented during the delay period to enable us to assess the resistance of neural representations in phonological WM to the distracting information. In a second experiment, with the same group of participants, we used TMS to directly test the causal role of the speech processing region (i.e., the left STG) and the putative buffer region (i.e., the left SMG) in phonological WM. The region that is crucial to maintaining phonological WM representations would be disturbed by TMS applied during the delay period of the phonological WM task and hence behavioral performance would be affected.

## Experiment 1: fMRI of Distractor Effects on WM

In early behavioral studies in the verbal domain, the Brown-Peterson task paradigm (Brown, 1958; Peterson and Peterson, 1959) was used to explore the effects of interpolated tasks on STM performance (e.g., recall; Crowder, 1976). In this paradigm, a short list of memory items (e.g., letters) is presented to subjects, followed by a short delay period filled with some distracting activity (e.g., reading aloud numbers during the delay or counting backward). Then the memory list items are recalled. Many studies have investigated the effects of the interpolated material on performance (Posner and Rossman, 1965; Crowder, 1967; Dillon and Reid, 1969). For example, Posner and Rossman (1965) found that a difficult interpolated task (e.g., judging if the sum of a pair of digits is odd or even) interfered more with memory performance than did an easy task (e.g., simply reading a pair of digits). One component of the interference from the interpolated tasks has been postulated to be a diversion of subjects’ attention from rehearsal of the memory stimuli, thus reducing recall of the memory list items (Peterson, 1969). Both buffer and embedded processes models include an attentional component [e.g., central executive in Baddeley et al. (2021) model; focus of attention in Cowan et al. (2021)], and thus an effect of diversion of attention is accommodated by both approaches. However, the effects of distractors cannot be accounted for solely on such grounds, as the degree of interference depended on the properties of the task-irrelevant materials *per se* (Wickelgren, 1965; Corman and Wickens, 1968; Landauer, 1974), specifically, the phonological similarity of the distractor items to the memory list items. For instance, Wickelgren (1965) found that recall of letters decreased as the number of phonologically similar interfering letters increased when subjects were required to write down the interfering items presented between the memory list and recall. Interference from the content of interpolated materials has been explained as overwriting of the memory items by the distracting items (Nairne, 1990). Such overwriting is assumed to be greater as the degree of similarity increases. In these studies, however, subjects were required to carry out a secondary task with the interpolated materials, and thus it is hard to attribute the interference solely to the interpolated materials automatically engaging WM due to perceptual processing of the stimuli, as the need to perform a task on the interfering material would necessitate that the information entered WM (Barrouillet et al., 2004). However, according to an embedded processes account, the interfering material should have an effect even if no task is required as that information should be processed in the perception region, causing interference with neural representations of the list items being maintained in that region. Another behavioral paradigm has examined the effects of irrelevant background speech on verbal WM performance, with detrimental effects on recall even though the irrelevant speech is to be ignored (see Neath, 2000 for a review). Unlike the effects of performing a task on interpolated material, the decrement from irrelevant speech does not depend on the phonological similarity of the background to the memory items and can be observed even with tonal stimuli (Jones and Macken, 1993). Also, the effect is typically demonstrated by presenting the irrelevant speech at the same time as the memory list items (which may be presented visually or auditorily), with effects of distractors presented after the set of memory items only occurring under certain conditions. The findings have thus led some to propose that the effect is an attentional one, rather than one due to a disruption of phonological storage (e.g., Jones and Macken, 1995). Recent behavioral studies continue to find mixed evidence regarding the explanation of the irrelevant speech effect in verbal WM, with some supporting the feature overwriting account (e.g., Oberauer and Lange, 2008) whereas others show that overwriting cannot explain a proactive interference effect in a memory list (i.e., earlier encoded items impact the recall of newly presented items which share features with those early items; e.g., Roodenrys et al., 2022).

More recently, neuroimaging approaches have been directed at assessing the effects of distractors on WM and the neural locus of such effects. Recent studies with multivariate approaches (e.g., multivariate pattern analysis, MVPA) have provided a way to assess the cortical response to distractors in the visual domain (Bettencourt and Xu, 2016; Lorenc et al., 2018; for a review see Lorenc et al., 2021). As compared to the traditional univariate neuroimaging approach, MVPA determines activation patterns associated with a few stimulus conditions or different features of items and has been regarded to be more sensitive in detecting WM storage representations (Sreenivasan et al., 2014; Sreenivasan and D’Esposito, 2019; though see Naselaris and Kay, 2015). For instance, Bettencourt and Xu (2016) found that while visual WM representations for grating patterns could be decoded in both the visual processing cortex and a proposed visual buffer region in the parietal lobe (e.g., superior intra-parietal sulcus) during a delay period when no distracting stimuli were presented, such decoding was only possible in the parietal lobe but not sensory cortex when distraction from various types of irrelevant visual items was present during the delay. In addition, in-scanner behavioral performance was not affected by the presence of distractors. Based on these findings, the authors suggested that since sensory regions need to be available to process other incoming stimuli (e.g., distractors), storage in the parietal lobe is needed to maintain a representation during distraction in order to achieve successful WM performance. The results are more consistent with a buffer model, suggesting a central role of the parietal lobe as a neural substrate for a buffer in maintaining visual WM representations.

In the present fMRI experiment, we employed an RSA approach (Yue and Martin, 2021) and included a distractor manipulation analogous to that of Bettencourt and Xu (2016). As compared to MVPA, which associates a few stimulus conditions to neural activation patterns, RSA can be used to evaluate the representational correspondence between the neural activation patterns and theoretical predictions based on the phonological similarity of stimulus items, thus providing a more sensitive approach to determining the nature of maintained representations (Naselaris and Kay, 2015). We manipulated whether there were distractors during the delay period or not and used nonwords as both memory items and distractor items to avoid an influence from semantics (Yue et al., 2019). In a prior study (Yue et al., 2019), we found converging MVPA evidence for phonological WM during the delay period in a proposed buffer region (e.g., the left supramarginal gyrus, SMG), as well as some MVPA evidence in a speech processing area (e.g., the left superior temporal gyrus, STG). Although the evidence in the SMG was stronger than that for the STG, the nature of MVPA decoding evidence is still vague. For instance, in the Yue et al. (2019) study, the MVPA decoding assessed whether speech vs. nonspeech could be discriminated but could not determine the basis of this discrimination. More recently, using an RSA approach, Yue and Martin (2021) further examined the phonological WM codes maintained in the left SMG and confirmed that the decoding evidence was attributed to phonological representations. In the present experiment, using RSA with the nonword stimuli allowed us to test whether phonological decoding was possible and whether such evidence was affected by distractors. We focused on the effects of distraction on decoding from the left SMG and the left STG. According to the embedded processes account, if the left STG serves as the neural substrate for phonological WM storage, RSA decoding evidence would be observed during the delay period in this region. In addition, to achieve successful recognition performance, the presence of distractors during the delay period would have no effect on the neural representations in the left STG if this region was the sole neural substrate for phonological WM storage, or the neural representations in the left STG would be reduced by the presence of distractors though they are still decodable. In contrast, according to the buffer account, the neural representations during the delay in the left STG (if any) would be affected by the presence of distractors (e.g., being wiped out by the distractors), whereas the left SMG should maintain memory representations, to achieve the undisrupted memory performance, even if distractors were presented.

### fMRI Experiment: Materials and Methods

#### Participants

Ten subjects (18–22 years old, mean: 19.7 years old, six females) recruited from Rice University participated in this experiment. All subjects were native speakers and reported no hearing, neurological, or psychiatric disorder. Subjects signed consent forms according to procedures approved by the Rice University Institutional Review Board to participate in the fMRI experiment and received monetary compensation or credit toward course requirements for their participation.

#### Materials and Procedure

A set of 16 one-syllable nonwords were used as the target memory items in the fMRI experiment in both the no-distractor condition and the distractor condition, allowing us to assess the effect of distractors on the phonological WM representations of the target nonwords. The 16 nonwords were created using online text-to-speech software^2^ mimicking a female speaker of standard American English and recorded at a sampling rate of 22.05 Hz. All nonwords were matched for the average sound amplitude by using the software Praat^3^. The average duration of the nonwords was 648 ms.

**Figure 1:**
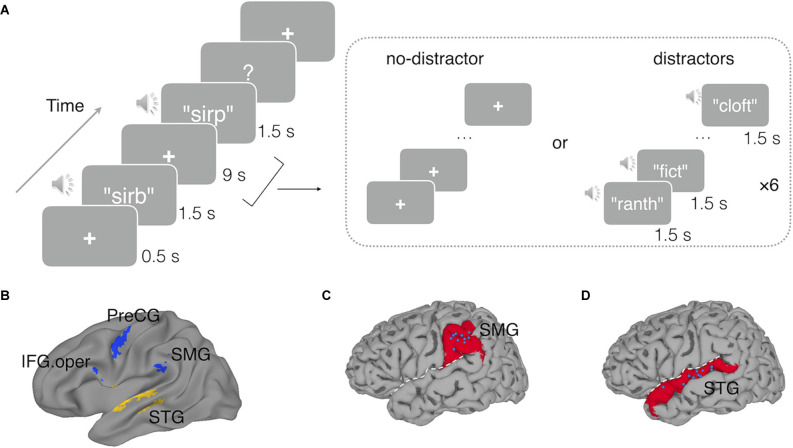
**(A)**An example trial with a non-matching probe in the delayed recognition task in the fMRI experiment. In the no-distractor condition, the delay period was silent with a fixation cross; in the distractor condition, the delay period was filled with six auditory distracting nonwords, presenting at a rate of 1.5 s per each. In the TMS Experiment, a similar delayed nonword recognition task was used except that a short duration of the delay period (3 s) was used. The memory nonword was presented for 700 ms, and triple-pulse TMS stimulation was triggered at either 700 ms (early) or 1,300 ms (late) after the onset of the memory nonword. **(B)** Functional regions of interest used in Experiment 1 derived from a prior study (Yue et al., 2019): the left superior temporal gyrus (STG), the left supramarginal gyrus (SMG), the opercular part of the left inferior frontal gyrus (IFG.oper), and the left precentral gyrus (PreCG). **(C)** The blue dots indicate the target sites stimulated by TMS in the left SMG and in **(D)** the left STG, with each dot representing a subject. White dashed line represents the Sylvain fissure. The underlying red regions mark the anatomical parcellations of the left SMG and left STG for reference.

A delayed recognition task was used (Figure 1). Each trial began with a fixation cross being presented for 500 ms in the center of a gray background screen. Then a spoken memory nonword was played to the subjects with a maximum duration of 1.5 s, followed by a 9-s delay period. Subjects were instructed to maintain this nonword over the delay period. Then a probe nonword was presented, and subjects were instructed to judge whether the probe nonword matched the memory nonword by pressing the left button if the probe matched the memory nonwords or the right button if not. There were two delay-period conditions. In the no-distractor condition, there were no other stimuli during the delay. In the distractor condition, the trial procedure was the same except that during the 9-s delay, a set of six distracting nonwords were presented at a rate of 1.5 s per each nonword. Subjects were instructed to remember the target nonword as their memory for that would be tested, but not for the delay period distractor stimuli. Thus, subjects just passively listened to the distracting nonwords without any explicit task. To reduce the potential confusion between the distracting stimuli and the memory items, the distracting nonwords were produced by a male speaker using the same software as for the memory nonwords. Previous studies have shown that speaker identities and phonemes are separately and independently represented in the human cortex (Formisano et al., 2008; Bonte et al., 2014), thus speaker identity information should not confound the ability to detect phonological representations (if any) of the memory nonwords which were of interest in this experiment.

For both the no-distractor and the distractor conditions, half of the probes were matching trials and half non-matching. The non-matching probes differed in a single distinctive feature of one phoneme from the target memory nonwords (e.g., sirb vs. sirp). For each trial, the distractor nonwords had no overlapping phonemes with the target memory nonword.

#### fMRI Procedure and Data Acquisition

In the fMRI experiment, the task was administered to subjects *via* E-prime 2.0 software (Psychology Software Tools^4^). The auditory nonwords were played binaurally *via* MRI-compatible earphones, and foam canal tips were used to reduce scanning noise. To ensure that subjects could clearly hear the nonwords and were aware of variation of phonetic features against the scanning noise, a short scanning session including 10 speech perception trials (i.e., discriminating pairs of nonwords which differ a single distinctive feature, “ba”-”pa”) was administered to each subject before the experimental functional scanning. The sound volume was adjusted to a comfortable level for each subject.

Each functional scan run contained trials from only one condition (either no-distractor condition or distractor condition), and in each scan, all unique 16 memory nonwords were played to subjects randomly with the inter-trial interval jittered at 4.5 s, 6 s, and 7.5 s. The average duration for each trial (stimuli presentation and post-trial interval) was 18 s. There were 9-s rest periods at the beginning of each scan, to allow equilibrium of the magnetic field, and at the end, to accommodate the hemodynamic delay of the last trial. The total duration for each functional scan was 306 s (5 min 6 s). There were six no-distractor runs and six distractor runs, with two types of runs being presented alternatively, and the order of two types of runs was counterbalanced across all subjects. There were 192 trials in total across the whole fMRI experiment for each subject, with 96 trials in the no-distractor condition and 96 in the distractor condition. Although the same memory nonwords were represented six times (across six runs) in both no-distractor and distractor conditions, the same memory nonword was never repeated within a run, and across six runs, the probe nonwords were never repeated. In other words, each time the subjects heard the same memory nonword, they encountered a new probe nonword.

The fMRI experiment was performed at the Core for Advanced Magnetic Resonance Imaging (CAMRI) at the Baylor College of Medicine. Images were obtained on a 3-Tesla Siemens Magnetom Tim Trio scanner (Prisma) equipped with a 64-channel head coil. Foam pads were used to keep subjects’ heads stabilized during the scanning. Functional scans were acquired by using a modified Massachusetts General Hospital Simultaneous Multi-Slice (SMS) EPI sequence which featured both high spatial and high temporal resolutions for the RSA approach with the following parameters: TR = 1.5 s, TE = 30 ms, FA = 72°, matrix size = 100 × 100, FoV = 200 mm, voxel size = 2 × 2 mm^2^. Each scan had 204 volumes and for each volume 69 2-mm thickness slices were acquired along the axial direction to cover the whole brain, with an SMS factor of 3. After the functional scans, an anatomical scan was also obtained with MPRAGE sequence: TR = 2,600 ms, TE = 3.03 ms, FA = 8°, matrix size = 256 × 256, voxel size = 1 × 1 × 1 mm^3^.

#### Data Analyses

##### Preprocessing

fMRI data preprocessing, general linear modeling and univariate group-level analysis were performed using AFNI software (version: AFNI_18.0.00; Cox, 1996). Preprocessing includes de-spiking of large fluctuation for some time points, slice timing, and head motion correction. The functional images were aligned to that individual’s anatomical image. The images were kept in the native space for the RSA approach and no spatial smoothing was applied to the data in order to preserve the spatial information across neighboring voxels. Spatial smoothing with a 4-mm full width half-maximum Gaussian kernel was applied to the functional data only for univariate activation analyses. A whole brain mask was generated and applied to the functional data, and voxel-wise signal scaling was calculated for each run. The resolution of functional data was kept in the native space with a voxel size of 2 × 2 × 2 mm^3^. For univariate voxel-wise group level testing, each subject’s data were warped to the Talairach standard space (Talairach and Tournoux, 1988) and registered to the TT_N27 template in AFNI.

##### General Linear Model

A general linear model was applied to the preprocessed time series to estimate the regression coefficients (i.e., beta values) for the no-distractor condition and the distractor condition respectively. For the RSA approach, a regressor was modeled for each individual memory nonword, with six repetitions across six runs for that nonword. Thus, 16 regressors of interest for 16 nonwords were included in the regression model. For the univariate analyses, a single regressor was modeled across all nonwords and all runs. A multiple parameter shape-free hemodynamic response function model (i.e., “TENT” function in AFNI) was used for each regressor to estimate the amplitude of signal change at each time point across the whole period for a trial (i.e., from the onset to 21 s later). The correct and incorrect trials were modeled separately, and all the following analyses were based on correct trials. Besides the experimental regressors, some nuisance regressors, including third-order polynomial baseline trends, six head motion correction parameters, and six temporal derivatives of head motion parameters, were also modeled. To reduce the influence of potential outliers, censoring was applied in the general linear model to the time points in which head motion exceeded a distance (i.e., Euclidean norm) of 0.3 mm with respect to the preceding time point or in which more than 10% of whole brain voxels were regarded as outliers by AFNI 3dToutcount. According to our calculation, on average across subjects, there were only 5.3 volumes (i.e., TRs; out of 2,448 across 12 runs, 0.22%) detected by 3dToutcount as outliers and censored out, indicating only a small proportion of outliers in the data.

##### Univariate Activation Analysis

For the group-level univariate analyses, the average amplitude of responses at the third and the fourth TRs (4.5 s and 6 s) after the onset of memory nonword was used as the signal change for the encoding period, and the average amplitude of responses across the sixth and the seventh TRs (9 s and 10.5 s) after the onset of memory nonword was calculated as the signal change for the delay period. Paired *t*-tests were performed on the signal changes to compare the distractor and no-distractor conditions, as well as the single condition vs. fixation baseline, during the encoding and the delay periods, respectively. Multiple comparison correction was conducted to estimate the cluster size threshold based on a permutation approach with a voxel-wise *p*-value of 0.001 and then corrected at the cluster-wise α value of 0.05. This simulation approach has been shown to effectively control the false positive rate under 5% (Cox et al., 2017).

##### Representation Dissimilarity Matrix

The phonological representation dissimilarity matrix (RDM) was constructed using the same procedure as in Yue and Martin (2021). The RDM represents the pairwise distances among 16 memory nonwords. Specifically, pronunciations of sixteen nonwords were obtained from the Carnegie Mellon University Pronouncing Dictionary^5^ and phonological transcriptions were coded with a set of phoneme symbols (ARPAbet; Shoup, 1980). Then, we used Phonological Corpus Tools^6^ to estimate the phonological distance for each pair of nonwords. To do this, phonological transcriptions were first aligned so as to minimize the number of different phonemes between two strings. All phoneme segments were mapped into a phonetic feature space (Hayes, 2008), and the distance between two phoneme segments was calculated as the distance between their phoneme feature values (Allen and Becker, 2015)—that is, the distance between two identical feature values is 0, while the distance of two opposite feature values (e.g., voiced/unvoiced) is 1, and the distance between two feature values in case one of them is unspecified is set to 0.25. Then, the phonological distance between two nonwords was calculated by adding up distances between all phoneme segment pairs.

##### ROI-Based RSA

Two regions of interest (ROI) were chosen from a recent study on testing the buffer vs. the embedded processes accounts of phonological WM (Yue et al., 2019): one region in the left STG (Talairach coordinates: *x* = −57, *y* = −15, *z* = 2) which was involved in speech processing and the other one in the left SMG (Talairach coordinates: *x* = −53, *y* = −33, *z* = 24). Converging evidence from neuroimaging studies (Paulesu et al., 1993; Salmon et al., 1996; Yue et al., 2019) and brain damaged patient data (Martin, 2005; Paulesu et al., 2017) had suggested the left SMG as a phonological buffer with phonological representations being maintained in this region. Besides the left STG and the left SMG, we also conducted an exploratory analysis in another two ROIs uncovered in Yue et al. (2019): the opercular part of the left inferior frontal gyrus (IFG.oper; Talairach coordinates: *x* = −59, *y* = 5, *z* = 20) and the left precentral gyrus (PreCG; Talairach coordinates: *x* = −49, *y* = −7, *z* = 40), as these two regions, particularly the inferior frontal gyrus, have been suggested to play a role in articulatory rehearsal in phonological WM (Paulesu et al., 1993; Chein and Fiez, 2001).

ROI-based RSA was performed by using CoSMoMVPA toolbox (Oosterhof et al., 2016) in Matlab (R2018a, The MathWorks, Inc., USA). Masks of prior ROIs were obtained for each subject by using an inverse transformation from the standard space to each individual’s native space. The RSA procedure was conducted during the delay period for the no-distractor condition and distractor condition respectively. In each ROI, we estimated neural RDM by calculating the Pearson correlation distance (Haxby et al., 2001; Kriegeskorte et al., 2008) on the neural activation patterns across all voxels in that ROI for all pairs of nonwords. Before calculating the pair-wise neural distances, the data were centered across all conditions (i.e., subtracting the mean activation pattern of all nonwords; Diedrichsen and Kriegeskorte, 2017). Then, the neural RDM was compared with the theoretical RDM by computing Spearman’s rank correlation (Kriegeskorte et al., 2008). For group-level inference, Fisher r-to-z transformation was applied to the Spearman correlation coefficient, and one-sample one-tailed *t*-tests were conducted to test the average similarity index against zero for a single condition. In addition, paired-sample *t*-tests were performed to determine if the difference between the no-distractor condition and the distractor condition (i.e., the distractor effect) was significant.

### Results: fMRI Experiment

#### fMRI In-Scanner Behavioral Results

**Figure 2:**
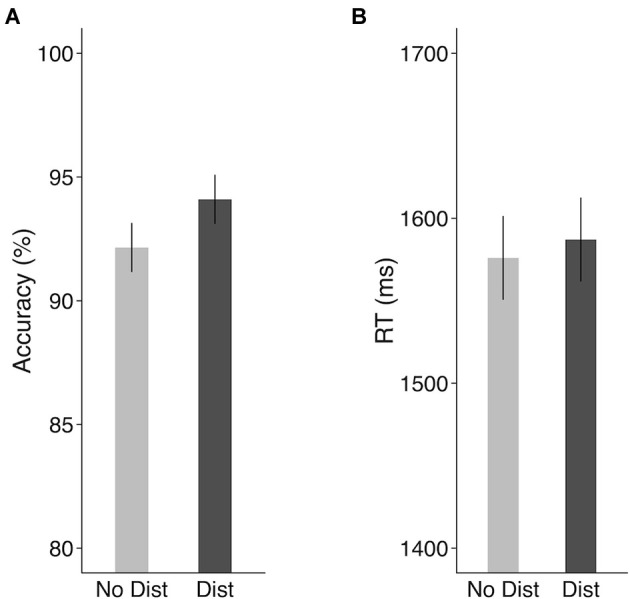
Behavioral results in the scanner in the fMRI experiment. **(A)** Accuracy and **(B)** response times. Error bars represent 95% within-subjects confidence intervals around the mean. No Dist, no-distractor condition; Dist, distractor condition.

As shown in Figure 2, distractors presented during the delay period did not decrease the recognition of the memory nonword (Accuracy: 92.2% for no-distractor condition and 94.1% for distractor condition). In fact, the presence of distractors marginally improved STM performance as compared to the no-distractor condition (*t*_(9)_ = 2.21, *p* = 0.054, Cohen’s *d* = 0.7, paired-sample *t*-test). Distractors had no effect on response times (1,576 ms for no-distractor condition and 1,587 ms for distractor condition, *t*_(9)_ = 0.49, *p* = 0.63, Cohen’s *d* = 0.15, paired-sample *t*-test). To correct for potential accuracy-RT trade-off effects and provide a better measure combining both RT and accuracy, we calculated an inverse efficiency (IE) score (i.e., the mean RT on the correct trials divided by accuracy; Townsend and Ashby, 1983). There was no significant difference in the IE scores between the no-distractor condition (mean: 1,717) and the distractor condition (mean: 1,693; *t*_(9)_ = 0.71, *p* = 0.49, Cohen’s *d* = 0.22). According to Nairne (1990) feature model of STM, a memory trace is susceptible to interference by an external list, with a feature in the memory trace being overwritten by a similar feature presented in the distracting list. It is possible that the lack of an interference effect resulted because of two factors: (1) no task was required for the distracting items; and (2) the distracting list had no overlapping phonemes with the memory nonword. The absence of distractor interference effect on behavioral performance suggested that features of the memory trace were preserved in the face of these unrelated distracting sounds.

#### Univariate Activation Results

**Figure 3:**
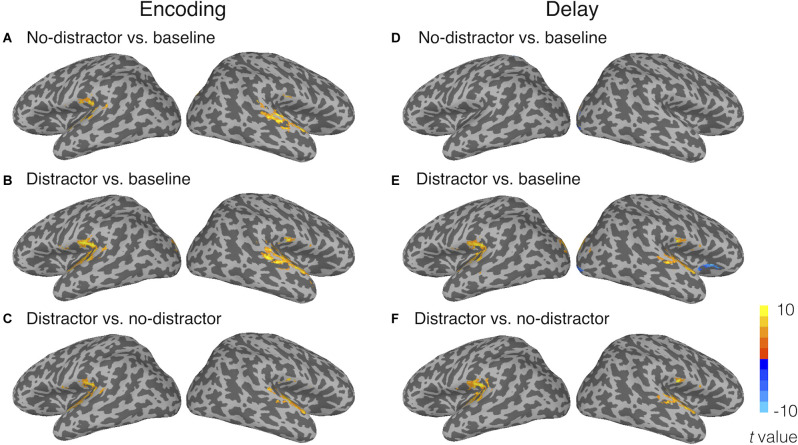
Univariate activation results during **(A–C)** the encoding period and **(D–F)** the delay period. Panels **(A)** and **(D)** show the activated regions for the no-distractor condition relative to the fixation baseline; **(B)** and **(E)** reveal the activated regions for the distractor condition relative to the fixation baseline; **(C)** and **(F)** show the contrasts of distractor vs. no-distractor conditions. The activation threshold was set at voxel-wise *p* < 0.001 and corrected at cluster-wise *α* < 0.05 (cluster size > 29 voxels).

Univariate activation analyses showed that, during the encoding period, the target memory nonwords similarly activated bilateral STG as compared to the fixation baseline in the no-distractor condition (Figure 3A) and distractor condition (Figure 3B). Because task activation was compared to a fixation baseline condition, it was unsurprising that the activated regions for perceiving nonwords included bilateral Heschl’s gyri (i.e., primary auditory cortex) and a large cluster in the right STG. The activated regions for the target nonwords also included the left supplementary motor area, the right superior occipital gyrus, bilateral cerebellum, and bilateral visual occipital gyri for both the distractor and no-distractor conditions (for all regions activated in the no-distractor and distractor conditions, see Supplementary Table S1 in Supplementary Materials). A contrast of distractor vs. no-distractor conditions during the encoding period showed that bilateral Heschl’s gyri and STG regions beyond Heschl’s gyri were activated more for the distractor condition than the no-distractor condition, suggesting that the distractors presented immediately after the memory nonword activated the primary and associated auditory cortex (Figure 3C). Given there was no jittering of the delay between the target nonword and distractors, it was not possible to strictly separate target and distractor activation.

During the delay period, no greater activity for the no-distractor condition relative to the fixation baseline was observed in the temporal lobe (Figure 3D). This is consistent with the univariate results from Yue et al. (2019) where no activation was uncovered in superior temporal regions during the delay period of a phonological STM task. Only a few clusters were observed in the right occipital lobes showing activity for the no-distractor condition relative to the baseline. In the distractor condition, during the delay period, greater activity relative to the fixation baseline was observed in bilateral STG, as well as in bilateral superior occipital gyri and bilateral lingual gyri (Figure 3E; for all regions activated in no-distractor and distractor conditions, see Supplementary Table S2 in Supplementary Materials). A contrast of distractor vs. no-distractor showed that bilateral STG and a cluster near the left SMG were activated more for the distractor condition than the no-distractor condition (Figure 3F). Greater activation during the delay period for distractor than no-distractor condition is likely due to two processes: perceiving the distractors and maintaining the memory word. Previous studies using a mismatch negativity paradigm (i.e., used as an index of unattended processing for task-irrelevant materials) have shown that automatic speech processing took place in the left temporal lobe (Auther et al., 2000; Tervaniemi et al., 2000; Pulvermüller et al., 2001; Saint-Amour et al., 2007). Thus, the activation in bilateral STG (basically in the primary auditory cortex) is no doubt due at least in large part to the automatic processing of distractors, whereas the activation in the left SMG might be due to maintenance of the memory word, considering the close location of this region (*x* = −45, *y* = −37, *z* = 22) to a region found in a recent study (*x* = −53, *y* = −33, *z* = 24; Yue et al., 2019). No region showed greater activity for the no-distractor condition than the distractor condition.

Taken together, univariate analyses showed that the memory nonwords activated similar brain regions during the encoding period either with or without distractors presented immediately after the memory nonwords. During the delay period, greater activity was found in bilateral STG for the distractor condition as compared to the no-distractor condition, which can be attributed at least in large part to the automatic activation evoked by the perception of the distracting nonwords. Greater activation in the left SMG is unlikely due to the perception of the distracting nonwords, as this region is not a typical speech processing region. Instead, the memory maintenance explanation for the activation in the left SMG is consistent with the notion that the left SMG plays a critical role in phonological WM in the face of distractors. However, without analyzing the neural representations for memory nonwords in the left SMG, it is hard to tell if this region truly maintained phonological representations in the face of distracting information. Also, the absence of activation in the left STG for memory nonwords during the delay period may just be due to lack of sensitivity with the univariate approach. Next, we performed RSA to address these questions.

#### RSA Results

**Figure 4:**
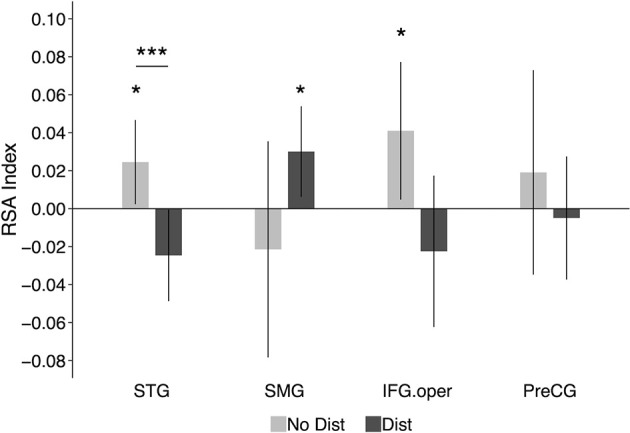
RSA results in functional ROIs defined based on results from Yue et al. (2019): the left STG, the left SMG, the left IFG.oper, and the left PreCG. The graphs show the average neural-model similarity index (i.e., Spearman correlation coefficient) during the delay period. Error bars represent 95% within-subjects confidence intervals around the mean. No Dist, no-distractor condition; Dist, distractor condition. STG, superior temporal gyrus; SMG, supramarginal gyrus; IFG.oper, inferior frontal gyrus (the opercular part); PreCG, precentral gyrus; *p < 0.05; ***p < 0.001.

Figure 4 shows the RSA evidence for phonological codes during the delay period in the distractor and no distractor conditions in the four ROIs: STG, SMG, IFG, and PreCG. In the left STG, there was significant RSA evidence of phonological coding in the no-distractor condition (similarity index_(mean Spearman rho)_ = 0.025, *t*_(9)_ = 2.02, *p* = 0.04, Cohen’s *d* = 0.64), but not in the distractor condition (similarity index < 0). In addition, a paired-sample *t*-test showed that the neural-model similarity index was significantly smaller for the distractor condition than the no-distractor condition (*t*_(9)_ = 4.70, *p* = 0.001, Cohen’s *d* = 1.49). In the left SMG, there was no RSA evidence in the no-distractor condition (similarity index < 0). This was unexpected because this region has been regarded as a phonological buffer and was thus expected to show RSA evidence for phonological WM maintenance when no distractors were presented, as was observed in a recent study (Yue and Martin, 2021). However, when distractors were presented during the delay period, there was significant RSA evidence in the left SMG (similarity index = 0.03, *t*_(9)_ = 2.31, *p* = 0.02, Cohen’s *d* = 0.73). The difference between the no-distractor and the distractor conditions was not significant (*t*_(9)_ = 1.53, *p* = 0.16, Cohen’s *d* = 0.48). However, a repeated-measure ANOVA with distracting conditions (i.e., no-distractor and distractor) and regions (e.g., STG and SMG) as two within-subject factors showed a significant interaction (*F*_(1, 9)_ = 7.7, *p* = 0.02, partial η^2^ = 0.46), showing that the decoding of phonological representations for the memory items was greater in the distractor than the no distractor condition in the SMG whereas the reverse was the case in the STG. Neither the distractor main effect nor the region main effect was significant (*p*s > 0.8).

In the left IFG (opercular part), significant RSA evidence was observed in the no-distractor condition (similarity index = 0.041, *t*_(9)_ = 2.07, *p* = 0.03, Cohen’s *d* = 0.65), suggesting that this region was involved in maintaining phonological representations during the delay period in the absence of distractors. Given the nonwords used in the experiment, the RSA evidence in the left IFG may imply that subjects relied more on rehearsal to maintain the memory nonwords (i.e., maintaining articulatory codes). When distractors were presented during the delay period, there was no RSA evidence in the IFG (similarity index < 0; see Section “Discussion” below). However, the difference between the distractor condition and no-distractor condition was not significant (*t*_(9)_ = 1.75, *p* = 0.11, Cohen’s *d* = 0.55). In the left precentral gyrus, there was no significant RSA evidence with either the presence or absence of distractors during the delay period.

To summarize, although the left STG showed RSA evidence for phonological codes during the delay period in the no-distractor condition, such evidence was absent when there were distractors presented during the delay period. While the findings suggest that the STG may provide support to phonological WM when no distractors are present, stronger causal evidence would be obtained in a paradigm in which neural representations are disrupted during the delay period. In contrast to the STG, the proposed buffer region in the left SMG showed RSA evidence for phonological retention in the distractor condition, suggesting its critical role in maintaining phonological information under distraction. In Experiment 2, we addressed the necessity of these regions in supporting phonological WM using a brain stimulation method.

## Experiment 2: TMS

Previous TMS studies have investigated the roles of specific regions in verbal WM (Romero et al., 2006; Deschamps et al., 2014; Sliwinska et al., 2015). For example, Deschamps et al. (2014), by using repetitive TMS (rTMS), tested whether the supramarginal gyrus is involved in phonological processing or in verbal working memory. To tap phonological processing, they used a same/different judgment task for pairs of two-syllable auditory stimuli (either spoken words or pseudowords) with a short stimulus-onset-asynchrony between items in a pair, which makes minimal demands on verbal WM. To tap verbal WM they used an N-back task with a subset of the same auditory stimuli as in the phonological processing task. The results showed that rTMS delivered to the SMG had no effect on the same/different judgment task but did impair performance in the N-back task (i.e., causing more errors and slower response times). The results from this experiment are consistent with the buffer account claiming that the left SMG, which is not involved in phonological processing, supports phonological WM. However, given the complexity of the N-back task, it is unknown what specific functional component of WM was supported by the SMG. The purpose of the present experiment was to test TMS effects in both the speech processing region and the putative phonological buffer region. Off-line rTMS, presented prior to the behavioral experiment, as in Deschamps et al. (2014), is not preferable because if there is a TMS effect in the speech processing region, it would be unclear whether this effect is attributed to a disruption of perception or memory retention. Instead, we used an online non-repetitive TMS paradigm (triple pulse) in this experiment, which allowed delivery of the stimulation after stimulus presentation during the delay period of a phonological WM task.

Some studies have used single- or triple-pulse online TMS paradigms to examine the causal role of the occipital lobe in visual WM (Cattaneo et al., 2009; van de Ven et al., 2012; van de Ven and Sack, 2013; Rademaker et al., 2017; van Lamsweerde and Johnson, 2017). For example, in Cattaneo et al.’s (2009) study, subjects were asked to remember the clock hands in a visual WM task, and a single-pulse of TMS was delivered over the occipital lobe either at the start or the end of a 2,000 ms retention period. They found that TMS applied at the start of the retention period caused an interference effect on WM performance (i.e., longer response time for TMS vs. no-TMS conditions), but this interference effect was absent when TMS was applied at the end of the retention period. This timing-sensitive TMS effect has also been observed in other studies (van de Ven et al., 2012; van Lamsweerde and Johnson, 2017) which examined the contribution of the early visual cortex to visual WM. van de Ven et al. (2012) manipulated the onset of a single TMS pulse during the delay period of a visual STM task (i.e., 100 ms, 200 ms, 400 ms after the onset of the 150 ms presentation period for memory items) and included two memory load conditions (i.e., one and three memory items). They observed an interference effect on memory accuracy (i.e., lower accuracy for TMS vs. no-TMS conditions) when a single TMS pulse was delivered at the 200 ms timing condition, but such an interference effect was absent when the TMS pulse was delivered later (i.e., 400 ms). In addition, this time-dependent pattern was only observed in the high memory load condition. These interference effects were claimed to support the embedded processes account for visual WM, in which the visual WM representation was maintained in the visual sensory area and TMS delivered at this region caused interference to WM performance. However, taking the discrepancy between early vs. late timings of TMS pulse into consideration, some researchers suggested another explanation—specifically, the absence of the interference effect at the late delay period reflected a nonessential role of the visual sensory region in WM. That is, once the WM representation has been transformed and consolidated into the visual buffer, TMS to the visual sensory region did not affect WM performance (e.g., Barbosa, 2017; Xu, 2017, 2018).

In this experiment, we employed a triple-pulse TMS paradigm to test the causal roles of the cortical regions showing neural evidence of phonological maintenance in Experiment 1. We used the same set of subjects in Experiment 1, and their RSA-fMRI results were used to locate the target regions for the TMS application. Results from Experiment 1 indicated that the left STG showed RSA evidence for phonological codes during the delay period in the no-distractor condition, and the left SMG showed RSA evidence for phonological storage during the delay period in the distractor condition. These two regions were chosen as the target regions for the TMS experiment. An occipital region was selected as a control region. A phonological WM task was administered to subjects, and triple TMS pulses were delivered to each of the target regions (i.e., the left STG, the left SMG, and the occipital control region) during the delay period of the phonological WM task. To uncover the potential time-course of TMS effects, we also manipulated the timing of the TMS pulses (see “Methods” Section). Recently, using this triple-pulse TMS paradigm, we showed that TMS applied to the left superior temporal lobe disrupted behavioral performance on a speech perception task, confirming the causal role of the left STG in speech processing (Ramos-Nuñez et al., 2020). If the left STG serves as the neural substrate for phonological WM, as predicted by the embedded processes accounts, and the RSA evidence for phonological codes in the left STG from Experiment 1 truly reflects phonological maintenance, then applying TMS to this region during the delay period should cause interference in WM performance. In contrast, the buffer models predict additional regions beyond the speech processing region involved in maintaining information in WM. Thus, buffer accounts would predict that TMS delivered at the left STG region would not affect WM performance, whereas stimulation at the buffer region (SMG) would.

### TMS Experiment: Materials and Methods

#### Participants

The same 10 subjects from the Experiment 1 participated in the TMS experiment. Subjects signed consent forms according to procedures approved by the Rice University Institutional Review Board.

#### Materials and Procedure

A similar delayed recognition task was employed in the TMS experiment, except that a shorter delay period (3 s) was used, as compared to 9 s used in the fMRI experiment. Each trial began with a fixation cross shown in the center of a PC monitor for 500 ms. At the end of the fixation cross, a nonword was played binaurally *via* earbuds to the subjects for 700 ms, followed by a 3-s delay. Subjects were instructed to remember the nonword over the delay period. Then a probe nonword was played and subjects responded to the probe by pressing buttons indicating whether the probe matched the memory nonword. The task was administered using E-prime 2.0 (Psychology Software Tools^4^). During the delay period, the TMS pulses were delivered to the target or control brain regions (see Section “TMS Procedures” below). Subjects’ response times were recorded from the onset of the probe nonword.

One-hundred and twenty nonwords, including the 16 nonwords used in Experiment 1, were used as the memory items in the TMS experiment. The mean duration of the nonwords was 615 ms (range: 481 ms–698 ms). As in Experiment 1, the non-matching probes differed in a single distinctive feature of one phoneme from the target memory nonwords (e.g., sirp vs. sirb). Half of the trials had matching probes and half non-matching.

#### TMS Procedures

Before the TMS experiment, each subject’s anatomical and functional images were acquired in Experiment 1. For the TMS experiment, we used the Brainsight TMS Navigation system (Rogue Research Inc., Canada) to register each subject’s head to the anatomical image for that subject by using four anatomical landmarks (i.e., the tip of the nose, the nose bridge, left and right ear notches). The functional data were registered to the anatomical images, thus the brain areas that showed RSA evidence served as the target regions for the TMS experiment. For each subject, three target regions were located based on the center of mass of clusters in RSA results in the fMRI experiment. The anatomical parcellation from Freesurfer (Fischl et al., 2004) was used to help locate the relevant areas—that is, the search was conducted within the range of the anatomical masks for the corresponding regions. The first target TMS region of interest was in the left SMG, which has been argued to be a buffer area for phonological WM (Martin, 2005; Paulesu et al., 2017; Yue et al., 2019); the second one was in the left STG, which was regarded as the speech processing area (Turkeltaub and Coslett, 2010; Price, 2012; Yi et al., 2019). The last one was a control region, and was located in the posterior occipital lobe, which is a primary visual processing area (Murakami et al., 2015; Ramos-Nuñez et al., 2020). Specifically, considering the results of the fMRI experiment, the selection of the target regions followed these priority criteria: for the left SMG, (1) if RSA evidence of phonological retention during the delay period (with a threshold of voxel-based neural-model similarity index > 0.1) was observed in both distractor and no-distractor conditions, the overlapping cluster was chosen as the SMG target region; (2) or if no overlapping cluster between distractor and no-distractor conditions was found, a cluster showing RSA evidence in the distractor condition was considered first; and (3) or if none of (1) and (2), a cluster showing RSA evidence in the no-distractor condition was chosen. With these criteria, five subjects showed RSA evidence in a left SMG for both distractor and no-distractor conditions, and four subjects showed RSA evidence for the distractor condition only, and one subject showed RSA evidence in the no-distractor condition only. For the left STG, in addition to the criteria described above, RSA evidence during the encoding period in this region was also considered—that is, if a common region in the left STG showed RSA evidence during both the encoding period and the delay period, that region was considered first; otherwise, a region showing RSA evidence during the delay period was chosen. There were four subjects showing delay-period RSA evidence in the distractor condition and six subjects showing delay-period RSA evidence in the no-distractor condition, with one subject showing both and one showing neither. For the control region, a posterior occipital region showing no RSA evidence during either period was chosen. With these criteria, we were able to locate three target regions for all subjects. The Talairach coordinates of three target regions for all subjects are shown in Table 1. Notice that all target regions were defined in each individual’s native space, and the coordinates in the standard space are only provided for reference.

**Table 1:** Talairach coordinates of the target regions in the TMS experiment.

Subjects	Left STG			Left SMG			Occipital		
	x	y	z	x	y	z	x	y	z
s103	−58	−9.6	−1.5	−45	−55.8	50.4	−4.5	−102.8	8.6
s104	−65.2	−31.9	5.7	−61.5	−39.4	34.4	−13.3	−101.7	12.3
s105	−63.8	−17.5	−2	−60.5	−31.5	41.5	−12.5	−96.5	23.5
s106	−56.7	−31.6	6	−63	−39.7	37.1	−9.6	−94.1	14.4
s107	−65.1	−19.2	9.5	−59	−28.9	40.3	−8.3	−99.1	14.8
s108	−64.6	−23.2	9.7	−58.5	−31.9	41.6	−4.5	−98.5	2.5
s109	−55.3	10	−4.9	−62.2	−37.2	38.6	−7.6	−95.4	19.3
s110	−65.6	−13.5	4.7	−59.1	−37	41.3	−10.5	−100.4	3.4
s111	−64.7	−21.3	5.8	−58.5	−49.1	37.9	−10.6	−97.5	−0.2
s112	−67.3	−23.7	2.7	−66.5	−29.4	25.1	−10.4	−98.8	4.6
Mean	−62.6	−18.2	3.6	−59.4	−38	38.8	−9.2	−98.5	10.3
SD	4.3	12.1	4.9	5.6	8.7	6.4	3	2.7	7.8

In TMS trials, during the delay period, triple pulses presented at 100 ms intervals (10 Hz) were delivered to the target region using Magstim Rapid^2^ simulator system (Magstim Inc.) with the D70^2^ coil being placed perpendicularly to the subject’s scalp. For the early timing condition, triple pulses were triggered at 700 ms after the onset of the memory item (i.e., the start of the delay period), and for the late timing condition triple pulses were triggered at 1,300 ms after the onset of the memory item (i.e., 600 ms later after the onset of the delay period). In the no-TMS trials, the coil was placed in the same region, but no pulse was delivered. For each target region and each timing condition, there were 10 trials for the TMS condition and 10 trials for the no-TMS condition, with the order of these 20 trials being pseudo-randomized (i.e., no more than three consecutive TMS trials) and grouped in a block. Orders for three target TMS regions (i.e., SMG, STG, occipital gyrus) and two TMS timing conditions (i.e., early, late) were counterbalanced across subjects. The stimulation intensity was set to the motor threshold for each subject individually at the beginning of the experiment. Before the formal experiment, a short practice session including five TMS trials and five no-TMS trials was administered to subjects with the coil placed at the vertex of the brain, to familiarize subjects with the stimulation procedure.

### TMS Results

#### Accuracy

**Figure 5:**
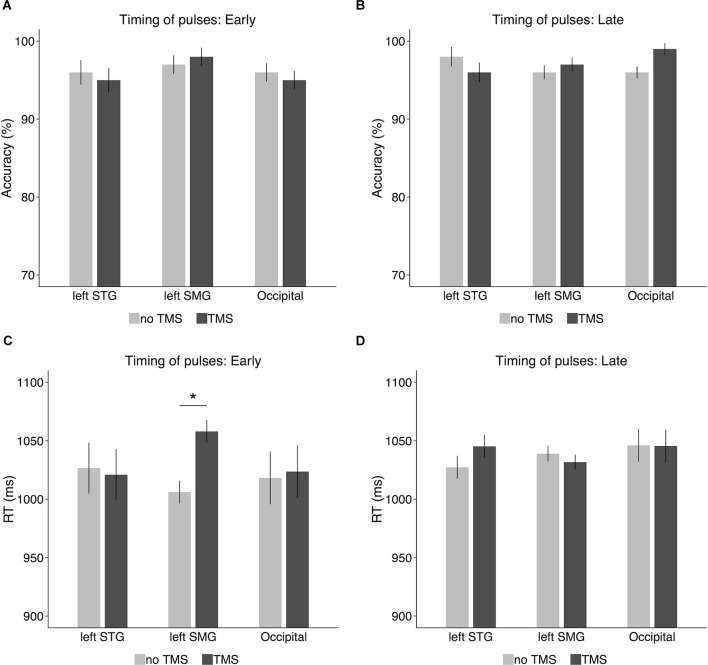
Accuracy and response times in the TMS experiment for **(A,C)** the early timing condition (i.e., the onset of the delay period) and **(B,D)** the late timing condition (i.e., 600 ms after the onset of the delay period). Error bars represent 95% within-subjects confidence intervals around the mean. *p < 0.05.

When the TMS pulses were delivered at the onset of the delay period (Figure 5A), there was no significant difference between the TMS condition and the no-TMS condition in either the left SMG (TMS: 98%, no-TMS: 97%; *t*_(9)_ = 0.43, *p* = 0.68, Cohen’s *d* = 0.14), the left STG (TMS: 95%, no-TMS: 96%; *t*_(9)_ = 0.32, *p* = 0.76, Cohen’s *d* = 0.1), or the occipital gyrus (TMS: 95%, no-TMS: 96%; *t*_(9)_ = 0.43, *p* = 0.68, Cohen’s *d* = 0.14). When the TMS pulses were delivered at 600 ms later than the onset of the delay period (Figure 5B), no significant TMS effect on accuracy was observed in either the left SMG (TMS: 97%, no-TMS: 96%; *t*_(9)_ = 0.56, *p* = 0.59, Cohen’s *d* = 0.18) or the left STG (TMS: 96%, no-TMS: 98%; *t*_(9)_ = 0.8, *p* = 0.44, Cohen’s *d* = 0.25). However, in the occipital gyrus, accuracy was slightly higher in the TMS condition (99%) than on the no-TMS condition (96%), a difference which reached marginal significance (*t*_(9)_ = 1.96, *p* = 0.08, Cohen’s *d* = 0.62).

#### RT

Response times were recorded from the onset of the probe nonword in the TMS experiment and analyzed on the correct trials^7^. As shown in Figure 5C, when the TMS pulses were delivered to the left SMG at the onset of the delay period (i.e., offset of the memory nonword), RT was longer for the TMS condition (1,058 ms) than for the no-TMS condition (1,006 ms). A paired-sample *t*test confirmed that the difference was significant (*t*_(9)_ = 2.69, *p* = 0.02, Cohen’s *d* = 0.85). However, this TMS effect on RT was not observed in the left STG (TMS: 1,021 ms, no-TMS: 1,027 ms; *t*_(9)_ = 0.13, *p* = 0.9, Cohen’s *d* = 0.04) or in the control region (i.e., occipital gyrus; TMS: 1,024 ms, no-TMS: 1,018 ms; *t*_(9)_ = 0.12, *p* = 0.91, Cohen’s *d* = 0.04). When the TMS pulses were delivered 600 ms later after the onset of the delay period (Figure 5D), there was no significant TMS effect on RT in the left SMG (TMS: 1,032 ms, no-TMS: 1,039 ms; *t*_(9)_ = 0.56, *p* = 0.59, Cohen’s *d* = 0.18), the left STG (TMS: 1,045 ms, no-TMS: 1,027 ms; *t*_(9)_ = 0.91, *p* = 0.39, Cohen’s *d* = 0.29), or the occipital gyrus (TMS: 1,045 ms, no-TMS: 1,046 ms; *t*_(9)_ = 0.02, *p* = 0.98, Cohen’s *d* = 0.006).

## Discussion

Although the buffer vs. embedded processes account of verbal WM have been under debate for decades, the neural locus of short-term retention and how the neural representations are maintained under distraction are poorly understood. In this study, we brought in evidence from two approaches to address these issues. Using fMRI with the RSA approach, we tested distractor interference effects on regions implicated in maintaining phonological representations and, using TMS, we tested the necessity of these regions on phonological WM performance.

If the representation for the memory nonword was maintained in the speech processing region (e.g., left STG), as predicted by the embedded processes account, the neural representation of the target would either persist despite the interference from distractors, or decrease but remain at a level from which the phonological representations were still decodable. However, this was not the case in the current study. Although RSA evidence for phonological retention was observed in the left STG in the no-distractor condition, such evidence was absent in the presence of distractors, and it was significantly different from that without distractors. Taking the lack of an interference effect on behavioral performance into consideration, this does not support a claim that the left STG, the speech processing region, is the neural substrate for phonological WM maintenance.

In contrast, the left SMG showed RSA evidence of phonological retention during the delay period in the distractor condition, supporting the role of buffer in this region in the face of distractors. One issue is that this region did not show RSA evidence of phonological retention when there were no distracting nonwords during the delay period. If this region serves as a buffer, it would be expected to maintain phonological information regardless of whether there were distractors or not. Given the present results, one might argue that the anatomical dissociation evident in the RSA evidence indicates that if there were no distractors, the phonological representations were maintained in the processing region whereas, in the presence of distraction, the phonological WM representations were shifted into a non-perceptual region. In a recent study on visual WM, Lorenc et al. (2018) presented a grating with a given orientation to the subject to remember, and then, for some trials, presented a distracting grating with a different orientation midway during the delay period. Using an inverted encoding model approach (a similar representation modeling method as RSA; Naselaris and Kay, 2015), they found that the orientation information of the memory grating could be successfully reconstructed from both visual sensory areas (e.g., V1-V3) and a parietal area (i.e., IPS) when there were no distractors. However, when a distractor was presented, the orientation representation in the visual sensory area showed a bias towards the distracting orientation whereas in the parietal lobe the representation did not show such a bias, and accurately maintained the memory grating orientation. Based on these results, Lorenc et al. proposed a dynamic trade-off mechanism between the processing region and the parietal region under different task demands (e.g., with or without distractors). However, this explanation does not seem to apply to our case. In the no-distractor condition in the present study, in addition to the left STG, RSA evidence of phonological retention was also observed in a posterior region of the left IFG. This frontal region has often been assumed to be involved in speech rehearsal (Paulesu et al., 1993; Awh et al., 1996; Chein and Fiez, 2001). Under this rehearsal view, one could reasonably assume that this region was involved in rehearsing the memory nonword during the delay period. The co-existence of RSA evidence in the left IFG and the left STG diminishes the possibility that the neural representations in the left STG underlie phonological WM. Instead, the decoding in the STG perhaps just reflects the automatic activation of phonological codes induced by inner speech rehearsal (Shergill et al., 2002).

However, there was a trend for RSA evidence in the left IFG to be modulated by distraction—that is, with RSA evidence with no distraction but not with distraction, although the difference was not significant. One might have expected that subjects would make more use of rehearsal to maintain the memory nonwords when distractors were present, but there was no suggestion of a higher decoding index in the IFG under distraction. However, it is not necessarily the case that RSA evidence in the left IFG should be interpreted as reflecting articulatory rehearsal. Some researchers have argued on the basis of behavioral results that participants do not tend to recirculate items through rehearsal in WM tasks and when instructed to do so, such rehearsal is not an effective means of maintaining information in verbal WM (Oberauer, 2019) and does not benefit performance (Souza and Oberauer, 2018, 2020). Chein and Fiez (2010) put forward a different interpretation of the role of the LIFG in verbal WM, arguing that this region was involved in maintaining novel sequences of phonological representations. If so, one might argue that these representations consist of output phonological representations involved in speech production (Martin et al., 1999; Cogan et al., 2017), rather than input phonological representations involved in speech perception, which might be maintained in the SMG. Chein and Fiez (2010) found that irrelevant auditory information presented during the presentation of the list and during a delay period decreased activity in this LIFG region and suggested that this resulted from a general effect of attention being oriented temporarily towards the irrelevant information. A related interpretation regarding the role of the left IFG is that it may be involved in another mechanism (e.g., refreshing; Barrouillet et al., 2004, 2011) which is used to maintain phonological representations. Thus, the absence of decoding in this region may reflect the diversion of attention by the distractors. It is possible that such a distraction effect occurred here, reducing the ability to decode information in this region, even though the behavioral performance was unaffected by distraction for our one-item memory load.

The findings from the fMRI experiment support predictions from a buffer account of phonological WM that non-perceptual fronto-parietal regions (i.e., the left SMG in the presence of distractors and the left IFG in the absence of distractors) are involved in maintaining phonological representations, although the left STG also showed decoding evidence in the absence of distractors. There are, however, some remaining issues. For example, it remained unclear whether the neural codes in the left STG in the no-distractor condition support phonological WM. We addressed this question by testing the necessity of the left STG, as well as the proposed buffer region (i.e., left SMG), in phonological WM with a brain stimulation approach. We did not observe any TMS effect on behavioral performance when stimulation was delivered to the left STG. In contrast, a TMS effect on response time was observed when stimulation was delivered at the left SMG at the start of the delay period. Because the left SMG showed RSA decoding evidence in the presence of distractors in Experiment 1 which is assumed to reflect WM storage rather than other processes involved in working memory (e.g., attentional control), and this region also showed neural evidence for phonological maintenance during the delay period without distractors in our recent studies (Yue et al., 2019; Martin et al., 2021b; Purcell et al., 2021; Yue and Martin, 2021), the TMS effect observed in this region serves as evidence supporting the necessity of this region in WM maintenance of phonological codes.

The discrepancy between early vs. late TMS effects in the left SMG makes the interpretation complicated. The early vs. late TMS timing manipulation was originally made to uncover potential time-dependent TMS effects in the processing region, which have been found in the visual WM domain (Cattaneo et al., 2009; van de Ven et al., 2012; Rademaker et al., 2017; van Lamsweerde and Johnson, 2017). However, no TMS effect on behavioral performance was observed in the left STG for either the early or late timing condition. Instead, time-dependent TMS effects were observed in the left SMG. If phonological representations were maintained in the left SMG over the delay period, TMS effects would be expected for both early and late timing conditions. The observed discrepancy may lead one to argue that such a TMS effect in the early timing condition might be due to some disruption of perceptual codes. This explanation seems implausible. If the early TMS effect is attributed to perceptual disruption, such a TMS effect should be observed in the left STG because this region is considered to be actually involved in speech perception, but no TMS effect was found. One explanation for the time-dependent TMS effect in the left SMG is that it may reflect the dynamic nature of WM codes in the buffer. In a recent neurophysiological study, Spaak et al. (2017) evaluated the generalization and dynamics of the single-electrode signal in a WM task recorded from the monkey prefrontal region. Specifically, they trained a decoder based on a given delay period and used this decoder to decode stimulus information during other delay periods. If the decoder shows good generalization in decoding information across different delay periods, the WM representation is assumed to be maintained in a stable state. They found that during the early delay period up to 500 ms after the offset of stimulus presentation, the neural codes for WM information changed dynamically, whereas, during the remaining delay period, the WM codes remained stable. A similar pattern has been observed in another neurophysiological study (Murray et al., 2017). Inspired by these observations, Barbosa (2017) proposed an explanation to reconcile the dynamic and stable nature of WM codes from a dynamic systems perspective—that is, when the sensory input disappears, the WM system evolves towards a stable state, but before that, the WM code remains dynamic and is vulnerable to external disturbance. Once the system achieves a stable state, distractors no longer have an effect on the WM representation. This claim seems to explain the discrepancy of the TMS effects in the present experiment. Immediately after the disappearance of the memory nonword and during the early delay period, the phonological information was being transformed from the speech processing region to the buffer region but the WM representation had not yet been constructed in the buffer, leading the WM code to be susceptible to interference (e.g., by the TMS pulses), but once the WM representation had been consolidated in the buffer, it remained in a stable state and external stimulation had a negligible effect on WM performance. Similar time-dependent interference effects on visual WM from behavioral data have been reported showing that performance on a visual arrays task was impaired by masks which were presented shortly after the memory arrays (e.g., less than 200 ms), but not when masks were presented more than 500 ms later (Vogel et al., 2006). As compared to the behavioral data, the present experiment shows that the neural locus of this interference effect on phonological WM is in the left SMG, implying its role in buffering phonological codes. Then, one question arises as to what kind of stable code is unaffected by TMS during the late delay period. This issue relates to the nature of WM (e.g., a distributed WM representation; Christophel et al., 2017). If the WM representation is maintained in a distributed manner along fronto-parietal regions, disturbance at one region within the fronto-parietal network may not sufficiently affect behavioral performance because a disruption of the neural representation in a local region may be restored or compensated *via* its connection from other regions in this network. This claim seems to be supported by the data from computational modeling work which showed that stronger functional connectivity between the prefrontal and parietal regions was associated with more stable memory representations (Edin et al., 2009; Constantinidis and Klingberg, 2016). If WM has a distributed nature, future studies using a multifocal TMS paradigm which applies TMS over two or more regions simultaneously are needed to test this claim (Hartwigsen et al., 2010). Also, future work using computational modeling approaches (e.g., Kowialiewski et al., 2021; Lemaire et al., 2021) should investigate the nature and functional properties of a phonological WM buffer.

It is possible that multiple non-sensory regions may be found to be involved in maintaining WM information, such as the left IFG implicated in the fMRI experiment. Results suggest that this region plays a different role in phonological maintenance (e.g., in maintaining input vs. output phonological codes; Martin et al., 1999; Cogan et al., 2017). If so, it is possible that the degree of disruptions differs across regions, providing some suggestion that the regions differ in their relative contributions in a given WM task. For example, a recall or a repetition task is assumed to rely more on the output phonological buffer than the input buffer. Then, performance on the recall or repetition task would be more impaired by TMS stimulations in this frontal region, as compared to TMS stimulations at the input buffer region in the inferior parietal lobe. Future work is needed to pin down the specific contribution of each region by TMS.

The present study with a non-repetitive TMS approach found a TMS effect on response time but not on accuracy. TMS-induced effects on high-level cognitive functions such as language and memory have usually been quantified by either change in RT and/or the accuracy of a given task (Hartwigsen, 2015). Accuracy effects (e.g., decreased accuracy for TMS condition vs. sham condition) on phonological WM have typically been reported in studies with a repetitive TMS approach (Romero et al., 2006; Deschamps et al., 2014), though RT effects have been observed in these studies as well. The triple-pulse TMS procedure used in the present is assumed to induce a short-lived suppression effect, as compared to the repetitive TMS that has a long-lasting suppression effect. Also, it should be noted that those studies which found TMS accuracy effects typically used phonological WM tasks that tapped phonological retention for multiple items (Romero et al., 2006; Deschamps et al., 2014). Thus, the absence of the TMS effect on accuracy might be due to the short-lived suppression effect or a relatively simple phonological WM task (i.e., maintaining the sounds of one item) used in the present study. Future work using a repetitive TMS procedure with pulses filling up the delay interval or using a long-term repetitive TMS prior to the behavioral task would be expected to reveal the accuracy effect.

### Limitations

One limitation of the current study is the limited sample size. Some work suggested that a minimum sample size of 12 subjects is required for RSA (Nili et al., 2014; Popal et al., 2019). Thus, the results reported in the current study should be treated with caution. Another limitation is the mixed evidence in the RSA decoding in the left SMG in Experiment 1. We did expect that RSA evidence would be observed in this region regardless of whether there were distractors or not, as we observed RSA decoding evidence for phonological retention for words in a recent study (Yue and Martin, 2021). The discrepancy might be due to the different materials and tasks used in the current study as compared to those in Yue and Martin (2021). Moreover, future work employing a typical list recall task with multiple items is needed to disentangle the different representations for the content of items and their serial order structure in the list (e.g., Fan et al., 2021). Also, with an explicit task on the distractors, future work can test whether an interference effect on the representations in the buffer region is associated with a decrement in WM performance.

## Conclusion

To summarize, although the speech processing region in the left STG showed RSA evidence of phonological retention for nonwords during the delay period, such evidence was absent when distractors were presented. In contrast, the proposed buffer region in the left SMG showed RSA evidence of phonological retention even in the presence of distractors during the delay period. In addition, a TMS effect on response time for a phonological WM recognition task was observed when the left SMG was stimulated during the delay period, whereas stimulations at the left STG and an occipital control region had no effect on behavior, confirming the causal role of the left SMG in phonological WM. Converging evidence from two approaches provides greater support for a buffer account of phonological WM over an embedded processes account, with the proposed buffer region in the inferior parietal lobe being suggested to play a critical role in maintaining phonological information under distraction. Future work using either functional connectivity or the multi-focal brain stimulation approach is needed to uncover whether the memory representations are maintained in local regions or are distributed across the cerebral cortex in a network, and how such a distributed WM might support a range of behavioral performance.

## Data Availability Statement

The raw data supporting the conclusions of this article will be made available by the authors, without undue reservation.

## Ethics Statement

The studies involving human participants were reviewed and approved by Rice University Institutional Review Board. The patients/participants provided their written informed consent to participate in this study.

## Author Contributions

QY designed the study, conducted the experiments, collected and analyzed the data under the supervision of RM. QY and RM wrote the manuscript and approved the submitted version.

## Conflict of Interest

The authors declare that the research was conducted in the absence of any commercial or financial relationships that could be construed as a potential conflict of interest.

## Publisher’s Note

All claims expressed in this article are solely those of the authors and do not necessarily represent those of their affiliated organizations, or those of the publisher, the editors and the reviewers. Any product that may be evaluated in this article, or claim that may be made by its manufacturer, is not guaranteed or endorsed by the publisher.

## Footnotes

^1^It should be noted that what we refer to as phonological processing in the current study involves those processes that map acoustic input to phonological long-term units (e.g., phonemes, syllables, words), but not some other general processes (from a broad perspective) that are involved in a task which relies on phonological retention. However, that is not to say that the role of the left SMG is exclusively as a phonological buffer. It may also be involved in other complex functions related to phonology (e.g., Jacquemot et al., 2003; Stoeckel et al., 2009; Church et al., 2011), but we focus on phonological maintenance in the current study.

^2^ http://www.fromtexttospeech.com

^3^ http://www.fon.hum.uva.nl/praat/

^4^ https://pstnet.com/

^5^ http://www.speech.cs.cmu.edu/cgi-bin/cmudict

^6^ http://phonologicalcorpustools.github.io/CorpusTools/

^7^In the main text, RT results are algebraic means. We also analyzed the RT data using both the median of response times and the mean of log-transformed response times (see Supplementary Materials). These two ways of analyzing response times gave similar results, consistent with what we reported in the main text.
