# Supplementary material for: Phonological Working Memory Representations in the Left Inferior Parietal Lobe in the Face of Distraction and Neural Stimulation
Source: Front Hum Neurosci. 2022 Jun 23;16:890483. doi: 10.3389/fnhum.2022.890483 (PMC9259857; doi:10.3389/fnhum.2022.890483)
Supplement: Supplementary file 1 [file Data_Sheet_1.pdf]

Supplemental Materials for

Phonological working memory representations in the left inferior parietal lobe in the face of distraction and neural stimulation

Qiuhai Yue[1,2], Randi Martin[1]

[1] Department of Psychological Sciences, Rice University, Houston, Texas 77005

[2] Department of Psychology, Vanderbilt University, Nashville, Tennessee, 37240

*** Correspondence:**

Qiuhai Yue (yueqiuhai@gmail.com)

Randi Martin (rmartin@rice.edu)

Supplementary Tables

Table S1. Univariate activated regions during the encoding stages in the phonological working memory tasks with and without distractors.

Regions	Talairach Coordinates *x*	*y*	*z*	Peak *t* value	Cluster size (voxel)
No distractor condition vs. baseline					
Right cerebellum and occipital gyrus	9	-65	-12	11.52	552
Right superior temporal gyrus	55	-29	6	15.68	418
Left calcarine gyrus	-17	-63	6	11.04	289
Left superior temporal gyrus	-45	-25	12	10.20	190
Right cerebellum	21	-61	-42	8.39	54
Left putamen	-17	5	8	9.74	50
Right cuneus	11	-91	16	8.99	45
Left cerebellum	-9	-69	-12	7.83	44
Left supplementary motor area	-7	1	60	11.82	41
Right superior occipital gyrus	19	-77	24	6.49	39
Left superior temporal gyrus	-59	-43	12	8.33	37
Left superior temporal gyrus	-61	-7	0	7.92	29
Distractor condition vs. baseline					
Right superior temporal gyrus	55	-31	6	20.52	927
Left superior temporal gyrus	-45	-21	12	18.78	697
Right superior occipital gyrus	9	-91	16	9.66	291
Left lingual gyrus	-13	-57	-4	7.72	84
Left supplementary motor area	-7	7	50	7.88	63
Right calcarine gyrus	11	-61	8	7.19	62
Right cerebellum	19	-61	-20	8.83	50
Left cerebellum	-29	-53	-20	7.46	46
Right cerebellum	19	-61	-46	9.88	40
Right lingual gyrus	21	-55	0	7.37	38
Distractor vs. no-distractor					
Right superior temporal gyrus	57	-17	10	13.43	443
Left superior temporal gyrus	-43	-31	10	16.08	387
Right superior temporal gyrus	61	-31	12	8.58	41
Left superior temporal gyrus	-61	-35	16	6.63	30

Note: Results are reported based on a threshold at voxel-level $p<0.001$, and corrected at cluster-level $\alpha<0.05$, with cluster size > 29 voxels. Regions of peak activation are labeled according to the Talairach-Tournoux Atlas in AFNI.

Table S2. Univariate activated regions during the delay stages in the phonological working memory tasks with and without distractors.

Regions	Talairach Coordinates *x*	*y*	*z*	Peak *t* value	Cluster size (voxel)
No-distractor condition vs. baseline					
Right superior occipital gyrus	13	-91	16	8.62	48
Right lingual gyrus	15	-67	-8	9.22	34
Distractor condition vs. baseline					
Right superior temporal gyrus	55	-23	6	10.12	569
Left superior temporal gyrus	-41	-19	12	16.86	383
Right superior occipital gyrus	23	-89	26	11.23	233
Left superior occipital gyrus	-13	-93	16	14.95	156
Left superior temporal gyrus	-59	-25	10	9.17	53
Right superior occipital gyrus	9	-83	36	9.49	42
Left lingual gyrus	-15	-55	-2	7.78	38
Right lingual gyrus	17	-67	-8	9.60	34
Right lingual gyrus	13	-55	-6	7.43	34
Left Rolandic operculum	-53	-3	6	7.18	31
Distractor vs. no-distractor					
Right superior temporal gyrus	53	-19	12	12.67	574
Left superior temporal gyrus	-41	-19	12	13.28	487
Left supramarginal gyrus	-45	-37	22	8.45	41

Note: Results are reported based on a threshold at voxel-level $p<0.001$, and corrected at cluster-level $\alpha<0.05$, with cluster size > 29 voxels. Regions of peak activation are labeled according to the Talairach-Tournoux Atlas in AFNI.

Supplementary analyses

RT results in the TMS experiment

In using the median of response times, when TMS was applied at the early delay period, there was no significant differences between the TMS and no-TMS conditions in either the left STG (TMS: 1011ms, no-TMS: 1004ms, t=0.2, p=0.8) or in the occipital control region (TMS: 991ms, no-TMS: 990ms, t=0.02, p=0.9). There was a significant TMS effect in the left SMG (TMS: 1018ms, no-TMS: 978ms, t=2.25, p=0.05). When TMS was applied at the late delay period, there was no significant TMS effect in the left STG (TMS: 1055ms, no-TMS: 1007ms, t=1.45, p=0.18), the left SMG (TMS: 991ms, no-TMS: 1025ms, t=1.31, p=0.2), or the occipital region (TMS: 1032ms, no-TMS: 1005ms, t=0.75, p=0.5). For the mean of log-transformed response times, when TMS was applied at the early delay period, there was no significant differences between the TMS and no-TMS conditions in either the left STG (TMS: 2.9955, no-TMS: 2.9995, t=0.2, p=0.8) or in the occipital control region (TMS: 3.0007, no-TMS: 2.9988, t=0.1, p=0.9). There was a significant TMS effect in the left SMG (TMS: 3.0112, no-TMS: 2.9898, t=2.65, p=0.03). When TMS was applied at the late delay period, there was no significant TMS effect in the left STG (TMS: 3.0175, no-TMS: 3.0038, t=1.1, p=0.3), the left SMG (TMS: 3.0034, no-TMS: 3.0101, t=0.91, p=0.4), or the occipital region (TMS: 3.014, no-TMS: 3.0097, t=0.31, p=0.8).
